# *Robinia pseudoacacia* poisoning in two horses: A case report

**DOI:** 10.17221/55/2024-VETMED

**Published:** 2024-11-21

**Authors:** Tereza Novotna, Eva Samonilova, Jaroslav Klan, Lucia Frgelecova, Anna Mala, Zdenka Svobodova, Zuzana Drabkova

**Affiliations:** ^1^Department of Animal Protection and Welfare and Veterinary Public Health, Faculty of Veterinary Hygiene and Ecology, University of Veterinary Sciences Brno, Brno, Czech Republic; ^2^Equine Clinic, Faculty of Veterinary Medicine, University of Veterinary Sciences Brno, Brno, Czech Republic; ^3^Institute of Forensic Medicine and Toxicology, First Faculty of Medicine, Charles University Prague, Prague, Czech Republic; ^4^Department of Pathological morphology and Parasitology, Faculty of Veterinary Medicine, University of Veterinary Sciences Brno, Brno, Czech Republic

**Keywords:** bark, black locust, equine, hyperammonaemia, intoxication

## Abstract

This case report describes the poisoning of two mares from the same paddock with *Robinia pseudoacacia* (Black locust) bark. The poisoning manifested itself by the sudden onset of weakness and fever with transient improvement after the administration of non-steroidal anti-inflammatory drugs and fluids. After the initial stabilisation, the mares were left unattended overnight. One of them was found dead in the morning. The surviving mare developed colic and severe encephalopathy and had to be referred to the clinic. Blood tests revealed severe hyperammonaemia. After four days of symptomatic treatment, she fully recovered. *Robinia pseudoacacia* with bark freshly bitten off was found in the paddock. The necropsy of the dead mare revealed oedema of the brain and lungs, pleural haemorrhages, and hyperaemia and haemorrhages of the glandular mucosa of the stomach. The intestinal content was watery, without macroscopic findings of the bark. Due to the peracute onset in both mares at the same time, hyperammonaemia and paralytic ileus in the surviving mare, and the presence of *Robinia pseudoacacia* in the paddock, poisoning with this tree was highly suspected. The diagnosis was confirmed by the microscopic findings of *Robinia pseudoacacia* bark tissue in the gastric content of the dead mare.

*Robinia pseudoacacia* (Black locust) belonging to the family *Fabaceae*, is one of the most frequently found non-native species in Europe. It was the first North American tree species imported to Europe (Nicolescu et al. 2018). It is a fast-growing tree of high economic and cultural importance. On the other hand, it is an invasive species potentially toxic for humans and animals (Puchalka et al. 2021). Except from the inflorescences, all parts of the plant are toxic, especially the bark and seeds (Uhlig et al. 2008). The toxic compounds are the glycoside (robitin), the alkaloid (robinin) and lectins (robin, ricin, and phasin) (Vanschandevijl et al. 2010). Robin is one of the main toxins of this tree, its concentration is the highest in the bark and increases in the autumn months. The lectins may influence the intestinal permeability via interactions with epithelial cells. They exert their toxicity by the inhibition of the protein translation and agglutination of the erythrocytes, which leads to cell death and the subsequent disruption of the intestinal mucosal integrity (de Virgilio and Degryse 2014; Kocyigit 2023). Gastrointestinal irritation and ulceration often progress to haemorrhagic gastroenteritis. Ricin also disturbs the calcium homeostasis in the cardiovascular system, which may cause myocardial necrosis and cardiac haemorrhage. Despite horses being the animals most susceptible to its effects, *Robinia pseudoacacia* toxicosis occurs sporadically. Both gastrointestinal and central nervous system signs have been described in horses and usually occur one to two hours following ingestion. To date, the pathogenesis of the central nervous signs in black locust intoxication is not clear, but they may be attributed to the hyperammonaemia caused by a gastrointestinal disease (Uhlig et al. 2008; Vanschandevijl et al. 2010). Symptoms vary depending on the amount of toxin ingested. Colic signs dominate up to 70 g. The intake of larger amounts primarily leads to neurological symptoms of varying degrees. Laminitis is a potential complication (Uhlig et al. 2008). The treatment of choice consists of a gastric lavage and the administration of activated charcoal, balanced intravenous fluid therapy, nutritional support and ancillary sedation if necessary. The prognosis is favourable if the clinical symptoms are confined to the gastrointestinal tract (Uhlig et al. 2008; Poppenga and Puschner 2014). The final diagnosis is established on the basis of proof of *Robinia* bark in the contents of the stomach (Vanschandevijl et al. 2010). This study describes the poisoning of two mares by the bark of *Robinia pseudoacacia* located in their paddock. In one of the mares, the course was fatal, the other mare developed hyperammonaemic encephalopathy, but within four days of intensive therapy, she recovered completely. The diagnosis was subsequently confirmed by the microscopic findings of the bark tissue in the stomach content of the dead mare. To the best of our knowledge, this is the first described case of *Robinia pseudoacacia* poisoning in a horse in the Czech Republic.

## Case description

### HISTORY

**Figure 1:**
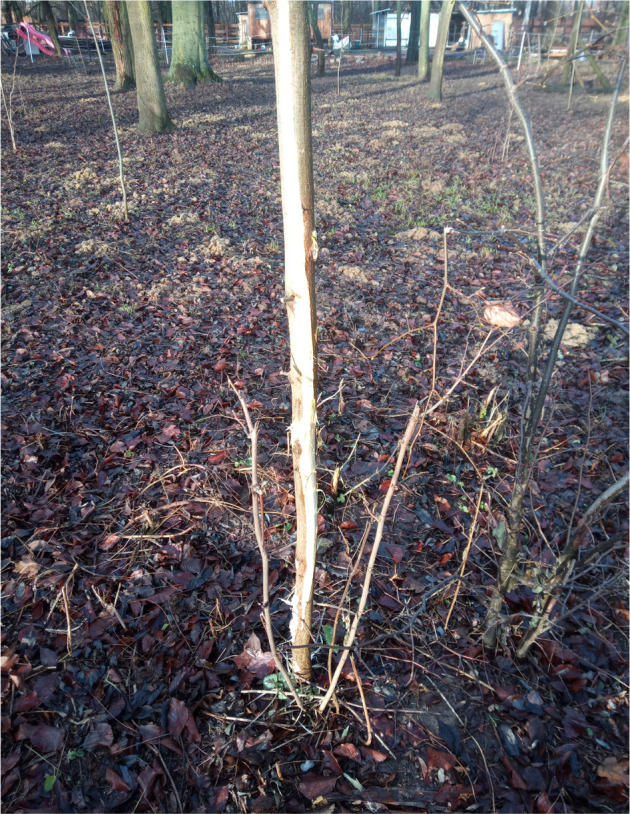
*Robinia pseudoacacia* with bark freshly bitten off in the paddock

Two Irish cob mares were found lying in the paddock with a fever (40 °C) and weakness in the afternoon. There was another asymptomatic mare in the same paddock at the time. A field veterinarian was called and administered non-steroidal anti-inflammatory drugs (NSAIDs) and fluids. The health status of both mares slightly improved after the therapy, but one of the mares was found dead the next morning. In the surviving 12-year-old mare, fever and weakness recurred and signs of mild colic (rolling, inappetence) developed. The field veterinarian sampled the venous blood for a haematological examination, which revealed leucopaenia with neutropaenia and lymphopaenia and mild monocytosis (Table 1). Antibiotics, NSAIDs and fluids were administered. Despite the treatment, the mare started showing ataxia and head pressing. Due to the rapid deterioration, she was referred to the Equine Clinic of the University of Veterinary Sciences Brno. A *Robinia pseudoacacia* tree with the bark freshly bitten off was later found during an inspection of the paddock (Figure 1). According to the owner, the mares had been kept in the paddock for four years already without any symptoms of poisoning. They spent the day in the paddock and they were in a stall during the night. There was hay available in the paddock and in the stall, and mineral licks were installed in the stall.

**Table 1:** Haematological analysis

Parameters	Values in the field	Values on the 3^rd^ day of hospitalisation	Reference ranges
Total erythrocytes (×10^12^/l)	9.47	8.49	6.2–10.2
Packed cell volume (l/l)	0.412	0.377	0.31–0.43
Haemoglobin (g/l)	149.0	135.0	111–159
Total leukocytes (×10^9^/l)	**2.43↓**	**10.03**↑	6.0–10.0
Neutrophils (×10^9^/l)	**0.92↓**	**6.32**↑	3.4–5.4
Lymphocytes (×10^9^/l)	**0.92↓**	**3.51↑**	2.0–3.2
Monocytes (×10^9^/l)	**0.44↑**	**0.1↓**	0.2–0.4
Eosinophils (×10^9^/l)	0.0	0.1	0–0.4
Platelets (×10^9^/l)	144.0	150.0	100–250

### CLINICAL COURSE AND OUTCOME

The mare was brought to the clinic in lateral recumbency, without any reaction to stimuli, and unable to stand up. She was sedated with xylazine at 0.5 mg/kg i.v. (Xylazin Ecuphar; Bioveta a.s., Ivanovice na Hané, Czech Republic) in the trailer and transported to the stable. The clinical examination revealed dark pink mucous membranes, a prolonged capillary refill time (CRT) of 3 s, an elevated heart rate (60 beats/min), a horizontal nystagmus, and the absence of gut sounds. A limited rectal examination on the recumbent horse revealed no abnormalities. Blood from the jugular vein was withdrawn for the acid-base status and biochemical examination. The results of the acid-base status examination showed severe metabolic acidosis, elevated lactate, hypokalaemia, hypocalcaemia and hyperglycaemia (Table 2). The biochemical examination revealed hypoalbuminaemia (27.5 g/l; ref. range 29–41 g/l), hyperbilirubinaemia (60.4 μmol/l; ref. range 13–34 μmol/l), slightly elevated aspartate aminotransferase (7.66 μkat/l; ref. range 1.7–5.83 μkat/l) and massive hyperammonaemia (207.3 μmol/l; ref. value < 60 μmol/l). An abdominal ultrasonography was very limited due to the lateral recumbency of the patient and only the left side of the abdomen was examined. The caecum was filled with fluid and some of the small intestinal loops were hypomotile with a fluid pattern. There was no oedema of the intestinal wall and no free fluid. Due to the rapid onset of the disease, paralytic ileus, neurological symptoms and hyperammonaemia associated with the presence of *Robinia pseudoacacia* with the bark bitten off in the paddock, poisoning by this tree was strongly suspected. Intravenous fluid therapy with a balanced crystalloid solution at 7 ml/kg/h i.v. (Plasmalyte; Baxter, Prague, Czech Republic) was started immediately. One litre of hypertonic saline at 3.3 ml/kg i.v. (7.5% NaCl; Sv. Anna Pharmacy, Brno, Czech Republic) was administered. Activated charcoal at 200 g *pro toto* p.o. (Aktivní uhlí; Dr. Kulich Pharma, Hradec Králové, Czech Republic) and lactulose at 250 ml *pro toto* p.o. (Lactulosa; Biomedica, Prague, Czech Republic) were dissolved in five litres of water and administered through a nasogastric tube. After stabilisation, the mare was able to stand up, but she showed severe head pressing and hyperflexion of the neck which led to dyspnoea. Due to the severe neurological signs, she was sedated repeatedly with detomidine at 0.01 mg/kg i.v. (Cepesedan; CP-Pharma Handelsges, Burgdorf, Germany) and kept in sternal recumbency. On the second day of hospitalisation, the health status of the mare rapidly improved, she was able to stand and she willingly moved around. There were no signs of colic or acute laminitis. The mucous membranes were still hyperaemic and mild dysphagia was evident. The acid-base parameters slightly improved, but the metabolic acidosis and hypokalaemia, mild hypocalcaemia and hyperglycaemia were still present (Table 2). The ammonia value was significantly lower (31.29 μmol/l; ref. value < 60 μmol/l). Based on the improvement of the clinical status and blood test results, the fluid therapy was discontinued. To correct the metabolic acidosis, a bicarbonate drink (100 g of natrium bicarbonate dissolved in 1 litre of plain water) was offered regularly. On the third day, the haematology was repeated and showed mild leucocytosis, neutrophilia, lymphocytosis and monocytopaenia (Table 1). On clinical examination, conjunctival hyperaemia was still apparent, but the mucous membrane of the oral cavity was pink. The intravenous catheter was removed and the mare was discharged from the clinic on the fourth day.

**Table 2:** Analysis of the acid-base status

Parameters	Values at admission	Values on the 2^nd^ day of hospitalisation	Reference ranges
pH	**7.257↓**	**7.267↓**	7.360–7.430
cK^+^ (mmol/l)	**2.9↓**	**2.6↓**	3.5–4.6
cNa^+^ (mmol/l)	140.0	142.0	136–142
cCa^2+^ (mmol/l)	**1.08↓**	**1.31↓**	1.40–1.70
cCl^–^ (mmol/l)	104.0	**115.0↑**	98–104
cGlu (mmol/l)	**9.4↑**	**9.0↑**	4.1–6.4
cLac (mmol/l)	**11.7↑**	1.7	1.0–2.0
Alactic base excess, c (mmol/l)	**–13.3↓**	**–11.5↓**	–2.0–4.0
Anion gap, c (mmol/l)	**23.4↑**	**13.4↑**	10.0–11.0

### NECROPSY

**Figure 2:**
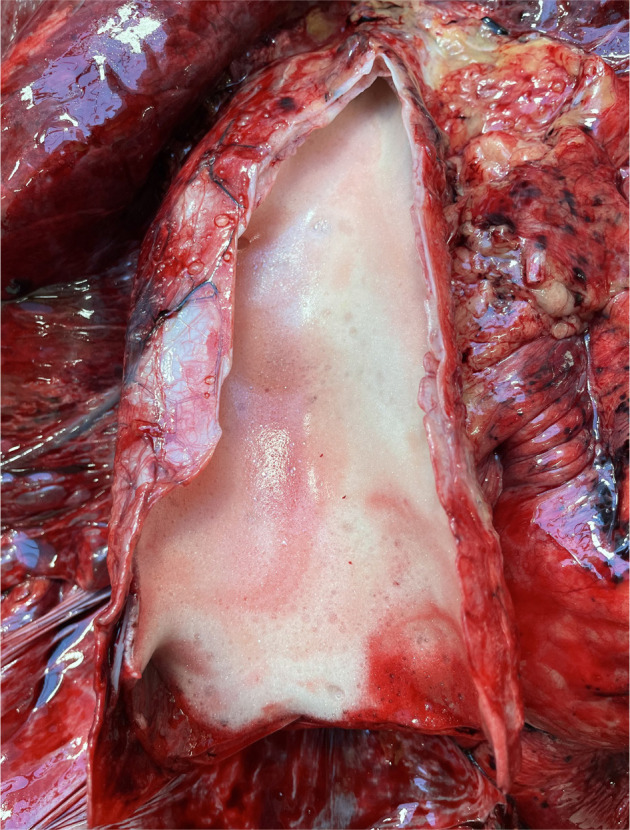
Pink frothy fluid in the trachea suggestive of the development of a pulmonary oedema

**Figure 3:**
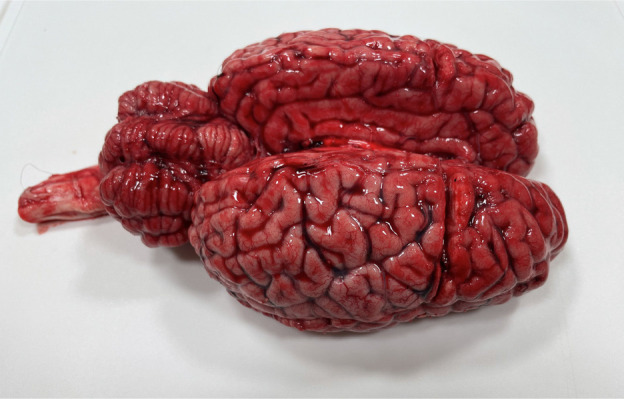
Congested and oedematous brain with fluid that accumulated in the convolutions and between the hemispheres

A necropsy was performed on the dead mare at the Department of Pathological Morphology and Parasitology of the University of Veterinary Sciences Brno, Czech Republic. Diffuse hyperaemia of the stomach wall and multiple haemorrhages on the glandular mucosa could be seen. The stomach was filled with partly digested feed and undigested straw, and no macroscopic pieces of bark were detected. The intestinal content was watery and pink, with pieces of undigested straw and fibrin strands. There was a pink frothy fluid in the trachea suggestive of the development of a pulmonary oedema (Figure 2) and multiple pleural haemorrhages were seen. The brain was congested and oedematous with fluid that accumulated in the convolutions and between the hemispheres (Figure 3).

A histopathological examination was not performed.

### TOXICOLOGICAL INVESTIGATION

**Figure 4:**
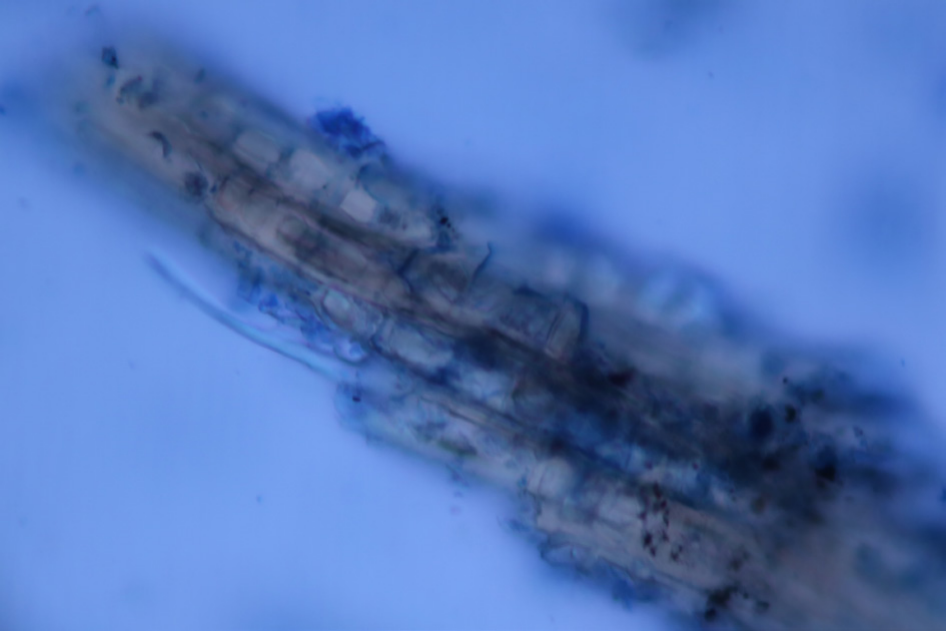
Bark tissue of *Robinia pseudoacacia* in the stomach contents. Inner bark consists of medullary rays with thin-walled cells, tubular and elongated in a radial direction (phloem)

The stomach contents were frozen at –80 °C and sent to the Institute of Forensic Medicine and Toxicology, First Faculty of Medicine, Charles University Prague, Czech Republic, for a toxicological investigation. The stomach contents were washed and filtered, the captured solid parts were checked under a binocular magnifier and then the individual fragments were examined under a microscope (Nikon Microphot FXA microscope at × 200–600 magnification). The anatomical structure of the covering tissue (periderm) of the tree *Robinia pseudoacacia* L. was demonstrated by a comparative method (Figure 4).

## DISCUSSION

This case report describes *Robinia pseudoacacia* poisoning in two mares from the same paddock at the same time. According to the literature (Cortinovis and Caloni 2015), poisoning by plants in horses may occur when forage alternatives are unavailable. In our case, there was hay available in the paddock. Probably, the mares became bored that day, which might have led them to chew the bark which resulted in toxicosis. Although this poisoning is considered rare, cases of horses poisoned with this tree have been reported in different European countries. In most of the cases, the horses ingested the bark, but poisoning with the roots, leaves, or branches has also been reported. In most of the cases, gastrointestinal signs manifested by colic of various intensities dominated, but central nervous signs and rarely laminitis and cardiovascular impairment have also been described (Sporri 1940; Schulze 1941; Keller and Dewitz 1969; Uhlig et al. 2008; Vanschandevijl et al. 2010). In one case of poisoning with freshly cut branches, stomatitis and hepatopathy were reported (Sporri 1940). Because no antidote exists, the treatment consists of the rapid decontamination and intensive supportive therapy aimed at the correction of the hypovolaemia, dehydration, acid-base status, and treatment of the gut inflammation. In the cases where central nervous symptoms develop, it is recommended to administer lactulose and possibly sedatives, which was undertaken in our case. The fever in both the patients probably resulted from an alimentary tract inflammation, which manifested later in the surviving mare by signs of colic and leucopaenia. Later, the severe central neurological signs connected with the massive hyperammonaemia probably resulted from the severe gut inflammation. The exact pathophysiological mechanism remains to be elucidated, but it is assumed that the increased ammonia production by bacterial overgrowth and/or increased ammonia absorption due to intestinal wall inflammation leads to hyperammonaemia. Additionally, haemorrhage into the bowel could probably also contribute to the hyperammonaemia associated with *Robinia pseudoacacia* intoxication in horses. Ammonia easily crosses the blood-brain barrier and high concentrations have a toxic effect on the neuronal cell membranes leading to typical signs of hyperammonaemic encephalopathy (Vanschandevijl et al. 2010). In the mare that died, according to pathoanatomical findings, severe encephalopathy probably developed during the night and resulted in a neurogenic pulmonary oedema. It is obvious from our case that the course of the poisoning differs individually. In one of the mares, the poisoning was fatal probably due to a higher dose of the bark ingested, but other factors, such as the age and general health status, could contribute to the progression of the disease. Based on the history, this 6-year-old mare was in good general condition. She was greedier, so she likely ate more of the bark, which led to a fatal course of poisoning. It is also described that young horses are more prone to poisoning (Caloni and Cortinovis 2015), probably due to their natural curiosity to taste something new and their lack of experience. Although developing similar clinical signs at the same time as the mare that died, the second mare survived without consequences. Based on this study, it can be seen that even if the poisoning with *Robinia pseudoacacia* has a peracute course with encephalopathy, with a fast and rapid intensive treatment and monitoring, the patient can be saved. The diagnosis is based on the history of the recent ingestion of the plant, the corresponding clinical signs, and the detection of parts of the plant in the content of the digestive tract. In our case, the poisoning was diagnosed based on the peracute onset in both mares at the same time, with the rapid development of the paralytic ileus and encephalopathy in the surviving mare. The *Robinia pseudoacacia* tree with bark freshly bitten off was found in the paddock where the mares were kept and although the pieces of bark were not seen macroscopically during the necropsy, they were subsequently detected on a detailed microscopical examination of the gastric contents, which confirmed the diagnosis.

## References

[R1] Caloni F, Cortinovis C. Plants poisonous to horses in Europe. Equine Vet Educ. 2015;27(5):269-74.

[R2] Cortinovis C, Caloni F. Alkaloid-containing plants poisonous to cattle and horses in Europe. Toxins (Basel). 2015; 7(12):5301-7. 26670251 10.3390/toxins7124884 PMC4690134

[R3] de Virgilio M, Degryse B. Harnessing the destructive power of ricin to fight human cancer. Ricin Toxin. 2014:208-37.

[R4] Keller H, Dewitz W. Poisoning of nine horses by the bark of false acacia, Robinia pseudoacacia. Dtsch Tierarztl Wochenschr. 1969;76:115-7. 5813869

[R5] Kocyigit E, Kocaadam-Bozkurt B, Bozkurt O, Agagunduz D, Capasso R. Plant toxic proteins: their biological activities, mechanism of action and removal strategies. Toxins (Basel). 2023 May 24;15(6):356. 37368657 10.3390/toxins15060356 PMC10303728

[R6] Nicolescu VN, Hernea C, Bakti B, Keseru Z, Antal B, Redei K. Black locust (Robinia pseudoacacia L.) as a multi-purpose tree species in Hungary and Romania: A review. J For Res. 2018 Nov;29:1449-63.

[R7] Poppenga RH, Puschner B. Toxicology. In: Orsini JA, Divers TJ, editors. Equine emergencies: Treatment and procedures. 4^th^ ed. St. Louis (MO): Elsevier; 2014. p. 268-88.

[R8] Puchalka R, Dyderski MK, Vitkova M, Sadlo J, Klisz M, Netsvetov M, Prokopuk Y, Matisons R, Mionskowski M, Wojda T, Koprowski M. Black locust (Robinia pseudoacacia L.) range contraction and expansion in Europe under changing climate. Glob Change Biol. 2021 Apr; 27(8):1587-600. 10.1111/gcb.15486 33336522

[R9] Schulze HG. Akazienvergiftung bei Pferden [Acacia poisoning in horses]. Tierarztl Wochenschr. 1941 Jan;6:65-6. German.

[R10] Sporri H. Poisoning of army horses by false acacia (Robinia pseudacacia). Schweiz Arch Tierheilkd. 1940;82:112-6.

[R11] Uhlig A, Grosche A, Hoops M, Schusser GF. Robinia pseudacacia (black locust) toxicosis in horses. Tierarztl Prax Ausg K Kleintiere Heimtiere. 2008;36(1):54-8.

[R12] Vanschandevijl K, Van Loon G, Lefere L, Deprez P. Black locust (Robinia pseudoacacia) intoxication as a suspected cause of transient hyperammonaemia and enteral encephalopathy in a pony. Equine Vet Educ. 2010 Jul;22(7): 336-9.

